# Isolation and characterization of nanometre aggregates from a Bai-Hu-Tang decoction and their antipyretic effect

**DOI:** 10.1038/s41598-018-30690-5

**Published:** 2018-08-15

**Authors:** Shaowa Lü, Hong Su, Shuang Sun, Yuyan Guo, Ting Liu, Yang Ping, Yongji Li

**Affiliations:** Key Laboratory of Ministry of Education, Department of Pharmacology, Heilongjiang University of Chinese Medicine, Harbin, 150040 China

## Abstract

In China, a decoction is one of the most common clinical dosage forms. Nanometre aggregates (NAs), which often consist of circular or irregular nanoparticles, have been observed in previous research on decoctions. A Bai-Hu-Tang (BHT) decoction is an ancient clinical dosage form in China. The purpose of this work was to isolate and characterize NAs from BHT and to investigate their antipyretic effect. A BHT decoction was prepared by the traditional method. The mechanism and active components of the aggregates in BHT were investigated by high-speed centrifugation, transmission electron microscopy (TEM), and HPLC (high-performance liquid chromatography). In addition to the aggregation, therapeutic activities were evaluated through temperature measurements, enzyme-linked immunosorbent assays, cellular uptake measurements and fluorescence imaging. The majority of the NAs in BHT had diameters of 100 nm, and the spherical structures contained C, O, Mg, Al, Si, Ca, Zn et al. Antipyretic bioactive compounds, such as neomangiferin, mangiferin, glycyrrhizic acid and ammonium glycyrrhizinate, existed in the aggregates. In addition, the NAs in BHT had a better antipyretic effect than the other dispersion phases of BHT. In particular, the nanometre aggregates of Bai-Hu-Tang (N-BHT) were easily taken up by cells, and the fluorescein isothiocyanate (FITC) signals of NAs were more enriched in the lungs and brain than in other organs over time. These results revealed that the antipyretic effect was associated with the NAs in BHT. The discovery of NAs might present a new perspective for understanding BHT decoctions and even lead to the development of a new nanomedicine approach in traditional Chinese medicine (TCMs). Therefore, this topic deserves further study.

## Introduction

In China, a decoction is one of the most common clinical dosage forms. The phytochemicals in herbal or plant materials are dissolved by boiling them in water. It is generally believed that the efficacy of insoluble phytochemicals is improved by the decocting process. However, so far, it is unknown in what form the bioactive constituents are dispersed and with what other components their delivery is achieved. Nanoparticles or aggregates that were naturally formed have been found in herbal medicinal broth^1^ and cooking soup^2^. Some primary tests were performed to determine their biological activities^3^. These results provided inspiration to determine the role of nanometre aggregates in decoction. Herbal or plant material is boiled to form decoctions, in which the chemicals are soluble and form hydrogen bonds, complexes and metal salts. The reason for this solubilization is that decoctions contain a variety of natural surface-active agents, such as saponins, sterols, gums, proteins and mucus, and micelles can be formed during the preparation process so as to solubilization. The physical and chemical properties of a decoction may also be influenced and changed by secondary metabolites, biological macromolecules and inorganic elements. In what form and with what components the bioactive phytochemicals are dispersed determine their delivery, assimilation, action and metabolism.

Related literatures have reported that TCMs form aggregates in aqueous solutions. Aggregation was observed in Ge gen decoctions^3^. Zhuang Y *et al*. demonstrated that aggregates commonly exist in TCMs and were able to survive the GI environment^1^. Nanometre aggregates can provide site-specific drug delivery via either a passive or active targeting mechanism^4^. Nanometre aggregates have a high solubilization capacity and targeting capability for poorly soluble Chinese herbal medicinal ingredients^5^.

BHT is a classic traditional Chinese herbal medicinal preparation that was first described in the Eastern Han Dynasty, approximately 1700 years ago. It has a good effect on the treatment of fever caused by acute infectious diseases such as pneumonia, epidemic haemorrhagic fever (EHF), influenza^6^ and acute suppurative tonsillitis^7^. BHT, composed of *Rhizoma Anemarrhenae*, *Radix Glycyrrhizae*, japonica rice and gypsum, is the classic prescription for high fever at the Traditional Chinese Medicine Hospital. *Anemarrhenae* is appointed as the principle ingredient, which is widely used for the treatment of fever and known for its anti-inflammatory and anti-diabetic effects^8^. The active component of *Anemarrhenae* is mangiferin. In addition, the main components of gypsum, japonica rice and *Radix Glycyrrhizae* are inorganic elements, polysaccharides, glycyrrhizins and ammonium glycyrrhizinate, respectively. A number of investigations were recently carried out internationally to identify the compounds relevant to the antipyretic effect of BHT. Wang *et al*. reported previously that gypsum alone can relieve fever, and its effect is rapid but brief; the effect of *Anemarrhenae* used alone is slow but persistent. When these two traditional Chinese medicines are combined, their pharmacological effects are better than the effects of the individual medicines^9^. Qiao Y *et al*. reported that mangiferin is one of the relatively insoluble constituents in *Anemarrhenae*; its effect is significant and may be related to the antipyretic mechanism of BHT^10^. Pharmacological studies indicate that mangiferin is related to anti-inflammatory, antipyretic, analgesic, antibacterial, immune regulation, hypoglycaemic and antioxidant effects^11^. As the principle ingredient of BHT, mangiferin provides pharmacological activities that are highly relevant to the therapeutic functions of the decoction. However, it was not entirely clear in what form mangiferin is distributed in BHT and how it is delivered to the target sites. Therefore, these questions are the major subject of this study. This study aims to analyse and elucidate the existing form of mangiferin in BHT and to investigate whether there are nanometre aggregates in BHT that improve the dissolution of mangiferin and increase its targeting ability.

## Results and Discussion

### Morphology of separated nanometre aggregates

**Figure 1:**
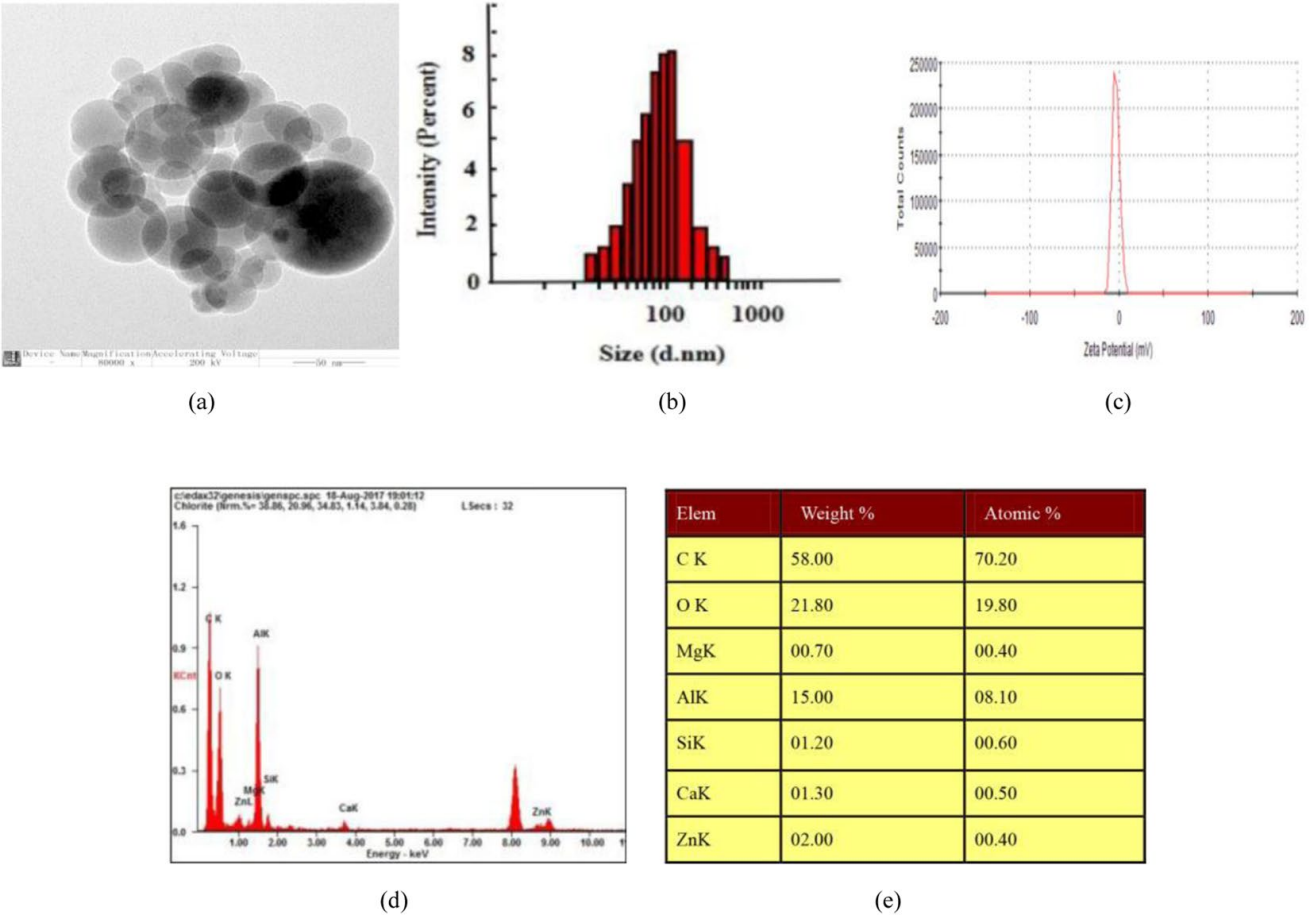
The characterization results for the nanometre aggregates in BHT. (**a**) TEM image of N-BHT showing spherical nanometre aggregates. In TEM, the image of NAs was magnified 40000 times. (**b**) Size distribution of N-BHT*. (***c**) The zeta potential of N-BHT (**d**,**e**) Elemental analysis of the spherical nanometre aggregates observed by TEM. The results indicated that the spherical nanometre aggregates contained C, O, Mg, Al, Si, Ca and Zn *et al*.

Colloidal nanometre aggregates from aqueous BHT extracts were separated and characterized by high-speed centrifugation coupled with dialysis. The morphological observations and nanometre aggregates were spherical and had different sizes (Fig. 1(a,b)), with diameters of 25 to 500 nm (majority at 100 nm). The electric potential of the nanometre aggregates was -3.11, and the polydispersity index (PDI) was 1 (Fig. 1(c)). The results of the elemental analysis of spherical nanometre aggregates contained C, O, Mg, Al, Si, Ca and Zn *et al*. (Fig. 1(d,e)).

**Figure 2:**
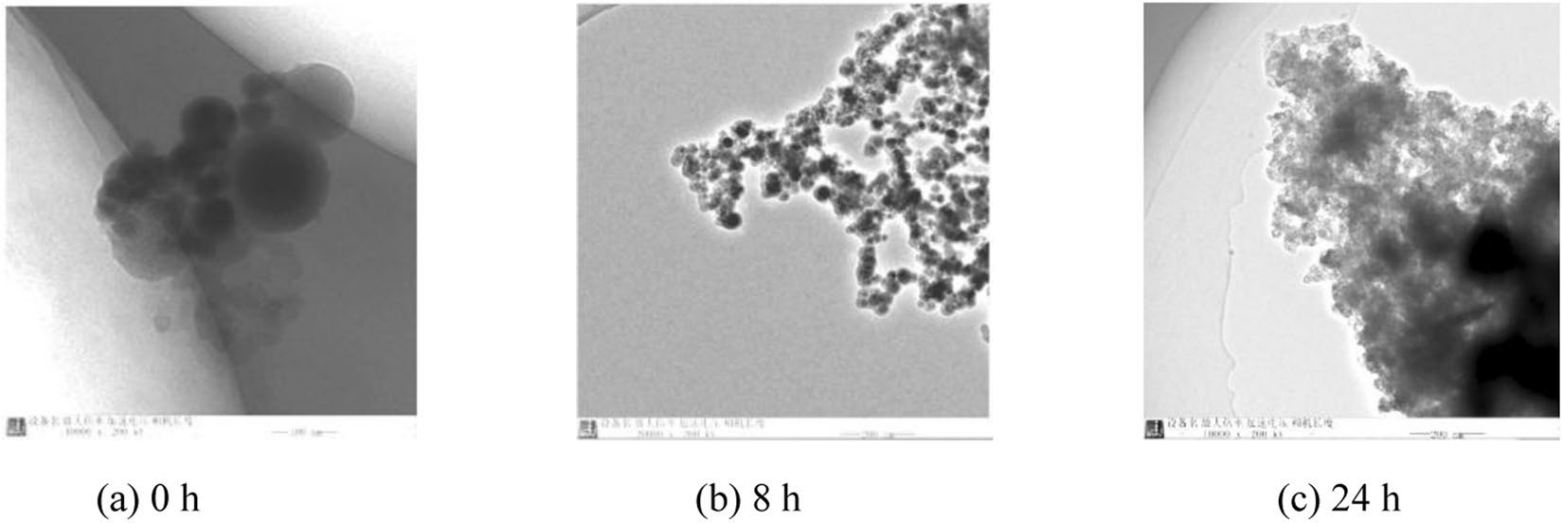
The stability of the NAs in BHT.

The results for the stability of NAs were shown that aggregated particles began to appear at 8 h. Over time, the aggregation increased (Fig. 2). This phenomenon may be caused by the NAs continuing to aggregate because of their small surface potential.

### Analysis of the neomangiferin, mangiferin, glycyrrhizic acid and ammonium glycyrrhizinate contents and distributions

**Figure 3:**
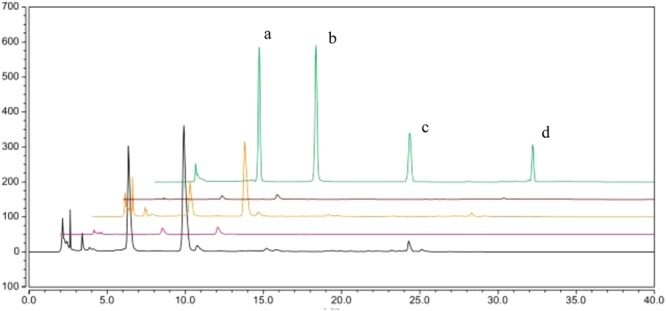
A representative HPLC chromatogram. From top to bottom: mixture of standard compounds, D-BHT, N-BHT, S-BHT and BHT. (**a**) neomangiferin; (**b**) mangiferin; (**c**) glycyrrhizic acid; (**d**) ammonium glycyrrhizinate.

Four phases were subjected to RP-HPLC to analyse the contents of neomangiferin, mangiferin, glycyrrhizic acid and ammonium glycyrrhizinate (Fig. 3). Their contents were linearly related to their peak areas over ranges of 0.27–3.12 μg·mL^−1^, 0.24–4.65 μg·mL^−1^, 0.07–0.60 μg·mL^−1^, and 0.02–1.20 μg·mL^−1^, respectively. However, the four different phases showed distinct variations in the abovementioned constituents. As shown in Table 1, in BHT, the total content of neomangiferin was 540.03 μg·mL^−1^; mangiferin, 1188.4 μg·mL^−1^; glycyrrhizic acid, 255.42 μg·mL^−1^; and ammonium glycyrrhizinate, 230.33 μg·mL^−1^. There was 483.00 μg of neomangiferin, 1068.88 μg of mangiferin, 219.93 μg of glycyrrhizic acid and 187.10 μg of ammonium glycyrrhizinate in 1 mL of N-BHT, which were obviously higher than the amounts in the other phases. The percentages of neomangiferin and mangiferin in N-BHT were 89.4% and 89.9%, respectively, of the total contents in BHT. These results indicated that mangiferin and neomangiferin were mainly distributed in N-BHT. The solubility of mangiferin in N-BHT was increased 10 times from 0.111 mg ·mL^−1^ to 1.068 mg ·mL^−1^, that is, nanometre aggregates contained more insoluble components. Moreover, it is unusual to detect neomangiferin, mangiferin, glycyrrhizic acid and ammonium glycyrrhizinate in the dialysis bag; all are small molecules with the molecular masses of 584.5, 422.3, 418.4, and 840.0, respectively, and it is usually considered that the dialysis bag is permeable to low-molecular-weight components. A reasonable explanation for this abnormal behaviour is that the abovementioned components were perhaps adhered or imbedded in the NAs of BHT.

**Table 1:** The contents of neomangiferin, mangiferin, glycyrrhizic acid and ammonium glycyrrhizinate (n = 3).

Compound	BHT (μg·mL^−1^)	N- BHT (μg·mL^−1^)	S-BHT (μg·mL^−1^)	D-BHT (μg·mL^−1^)
Neomangiferin	540.03 ± 10.36	483.00 ± 10.55	56.78 ± 4.90	2.10 ± 0.19
Mangiferin	1188.40 ± 1.04	1068.88 ± 9.67	74.22 ± 6.90	45.80 ± 3.55
Glycyrrhizic acid	255.42 ± 5.82	219.93 ± 7.08	24.18 ± 0.84	6.72 ± 1.53
Ammonium glycyrrhizinate	230.33 ± 7.79	187.10 ± 7.02	32.34 ± 2.60	0.87 ± 0.03

### Antipyretic effect

**Figure 4:**
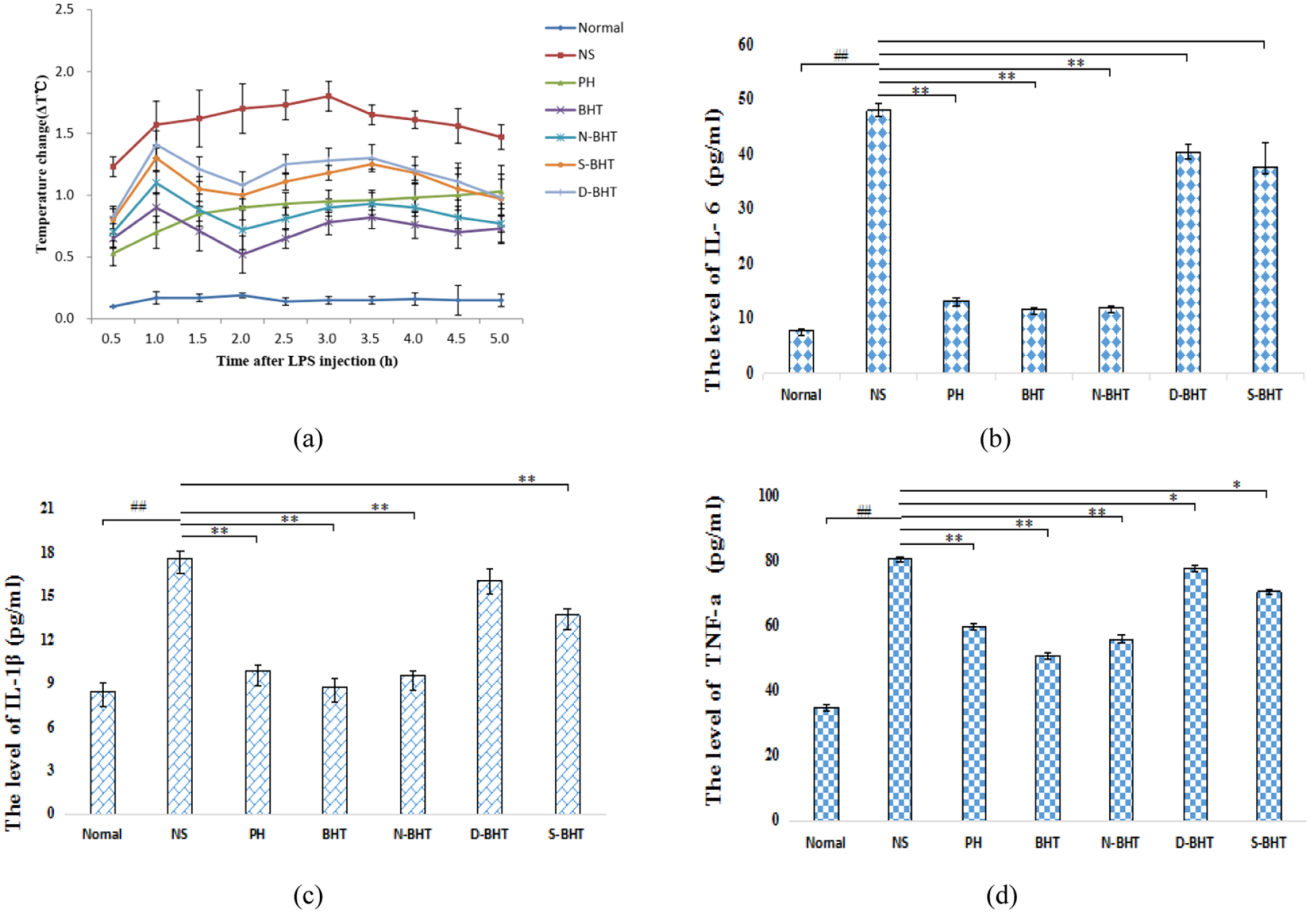
Effects of BHT, N-BHT, S-BHT, D-BHT and PH treatment on fever and plasma levels of IL-6, IL-1β and TNF-α in rabbits induced by LPS. The data presented are the average of 6 replicates (n = 6). Means with different symbols differ significantly: normal group vs. model ^##^P < 0.01; model vs. experiment group ^**^P < 0.01, ^*^P < 0.05.

Lipopolysaccharide (LPS) (20 μg·kg^−1^) significantly increased the temperature of model group rabbits, which exhibited curling up and trembling, and their body temperature (Tb) underwent a perceptible rise at 2 h after injection accompanied by a redness of the ears, eyes, and lips and a shortness of breath and reached a maximum at 2.5 h. Most rabbits suffered from febrile reactions such as cyanotic lips, conjunctival congestion, spirit depression, anorexia and thirst. In our study, we found that the Tb of the normal group essentially remained stable at all times, and the Tb of the model group showed a biphasic thermal response similar to that observed in other studies^12^. The peak fever temperatures were observed at 1 h (Δ1.57 ± 0.19 °C) and 3 h (Δ1.80 ± 0.12 °C), and the difference was statistically significant (P < 0.01) (Fig. 4a). Positive medicine control group of promethazine hydrochloride (PH) exhibited a marked antipyretic action, and the Tb of the PH group decreased at 0.5 h; however, its Tb increased after 0.5 h. Thus, PH was an effective but not long-lasting drug. In contrast, the antipyretic effect of BHT was effective and long-lasting. After 0.5 h, the difference between the Tb of the BHT group and the model group (all P < 0.05) was statistically significant (P < 0.05), and after that time, the Tb of the BHT group gradually decreased. The N-BHT group also exhibited significant differences from the model group from 0.5 h to 5 h, during which time the Tb gradually reduced (P < 0.05). The effect of N-BHT could last for 5 h, and its antipyretic effect on rabbits induced by LPS was the best. Both the dialysate of the Bai-Hu-Tang decoction (D-BHT) and the sediment of the Bai-Hu-Tang decoction (S-BHT) had weak antipyretic effects. These results demonstrated that the antipyretic effect of N-BHT was superior to that of the other parts of BHT. Thus, N-BHT was responsible for the antipyretic action of the effective phase of BHT. In our study, it was found that BHT at a concentration of 5 g·kg^−1^ could also be used as an alternative to reduce the effect of fever; in addition, this concentration is the routine clinical dosage and has also been used in previous studies^13^.

### Plasma levels of IL-6, IL-1β and TNF-α

After LPS induction, the levels in the model group increased significantly compared with those in the normal group (P < 0.01) (Fig. 4b,c,d). Administration of BHT and N-BHT significantly reduced the elevations of IL-6, IL-1β and TNF-α compared with the model group. However, compared with the effect of BHT, N-BHT showed the same effect—a reduction in the plasma levels of IL-6, IL-1β and TNF-α in rabbits; there was no significant difference between the two effects. The effects of D-BHT and S-BHT were not obvious.

#### Cellular uptake of nanoparticles

**Figure 5:**
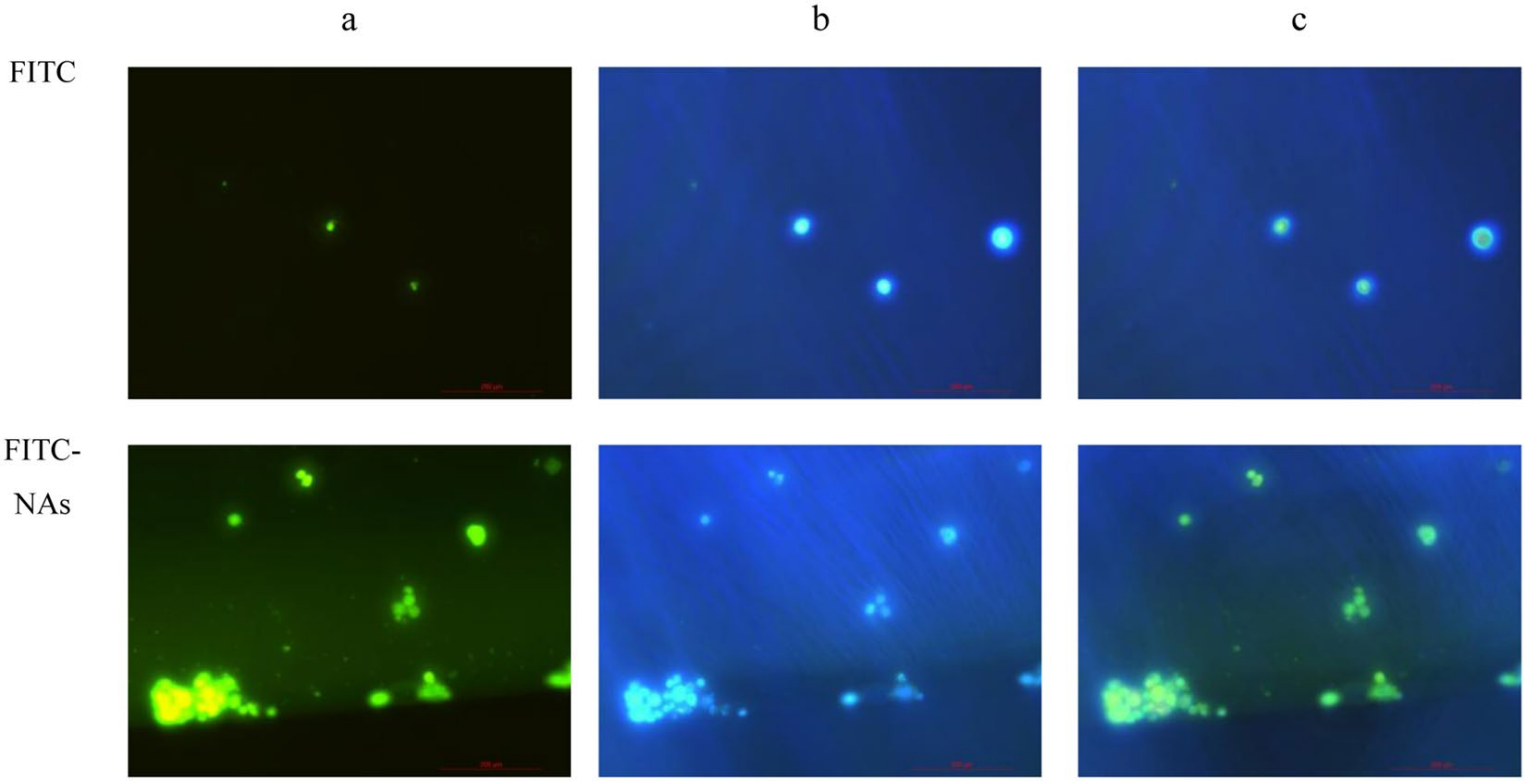
The fluorescence microscopy images of Caco-2 cells incubated with FITC-NAs (**a**) FITC channels showing green fluorescence. (**b**) DAPI channels showing the blue fluorescence from DAPI-stained nuclei. (**c**) Merged channels of A and B. Magnification ×200.

Fluorescence microscopy: The subcellular localization of free FITC in Caco-2 cells was investigated using fluorescence microscopy. The results indicated that only a small amount of FITC was observed in the nucleus of Caco-2 cells after 2 h of incubation with free FITC. The fluorescence signals of FITC-NA were observed in the Caco-2 cells, which were consistent with the results of flow cytometry (Fig. 5).

**Figure 6:**
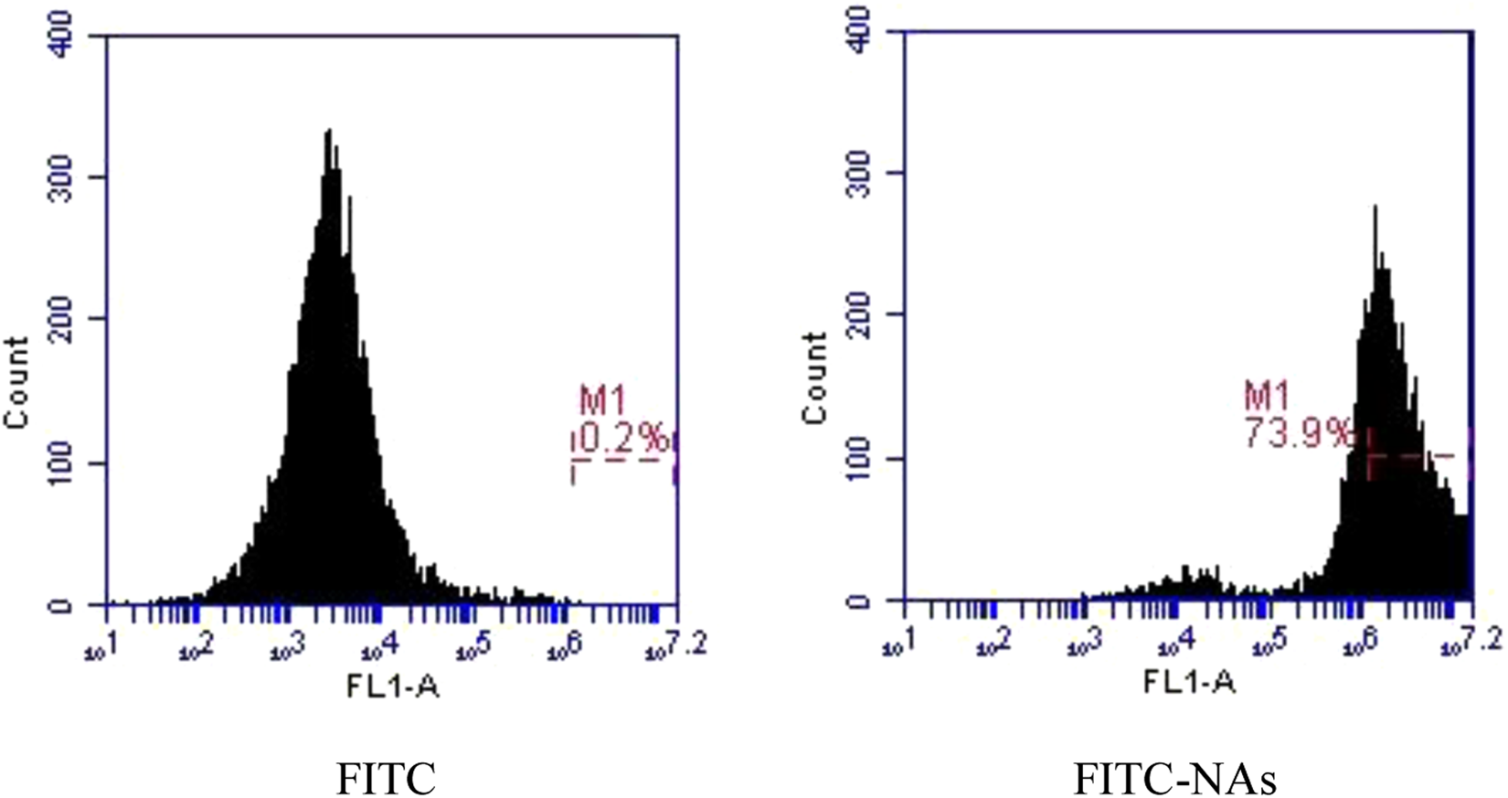
Results of the flow cytometry analysis of Caco-2 cells incubated with FITC-NAs 2 h post-treatment represented in dot plot graphs. Each measurement was repeated three times, and the number of events per reading was 20,000. The FL1 channel indicates the total percentage of the cell population emitting a fluorescence signal.

Flow cytometry: This study found a linear relationship between the fluorescence intensity and the amount of nanoparticles endocytosed by the cells. As illustrated free FITC as a control showed only auto-fluorescence (Fig. 6). Compared with free FITC, the relative cellular internalization of FITC-NA rose to 79.3%, suggesting a higher affinity for the cells. This is ascribed to the decrease in size, which results in easy access to the cells after the nanoaggregates had separated.

**Figure 7:**
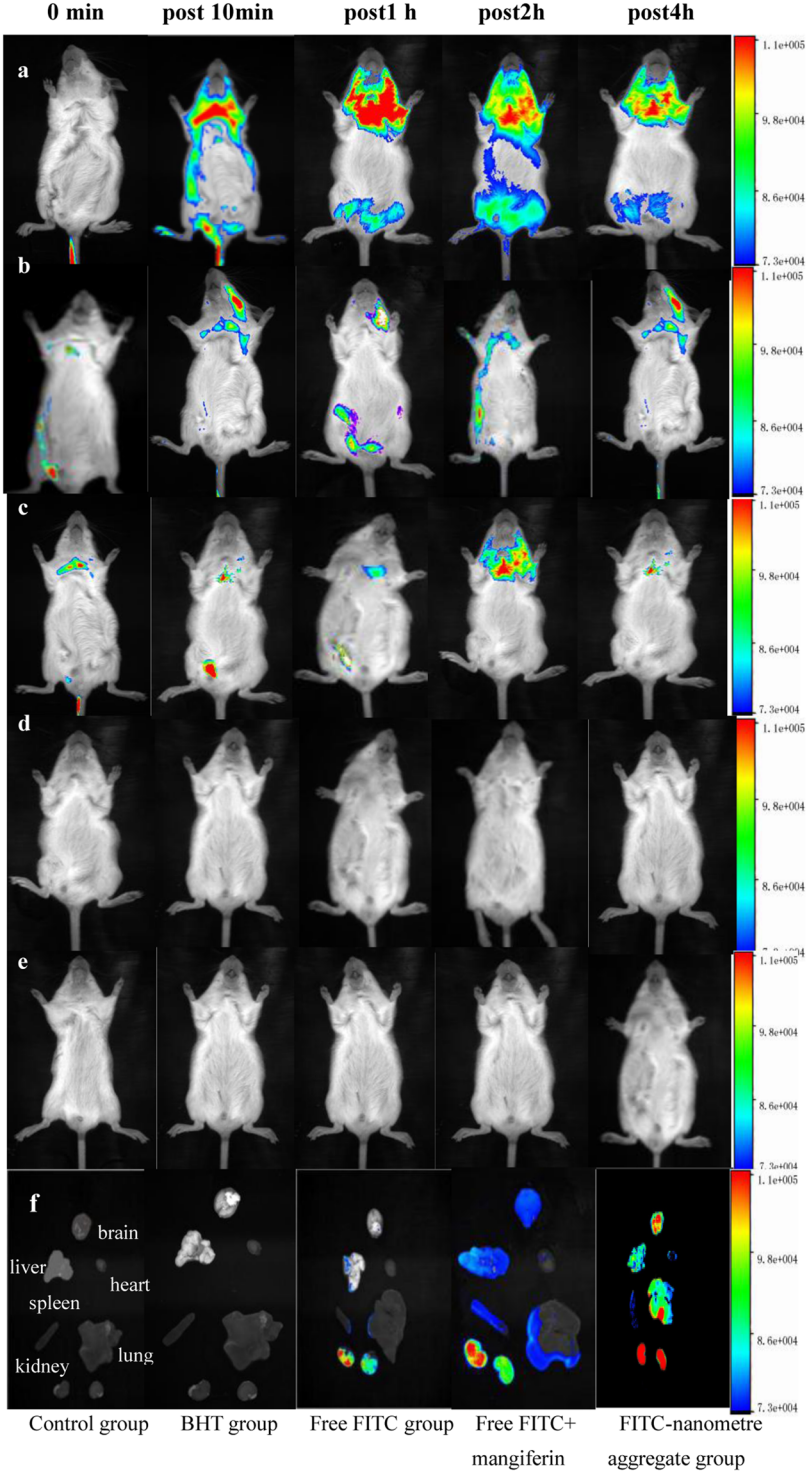
The fluorescence imaging of ICR mice (**a**) FITC-nanometre aggregate group; (**b**) free FITC + mangiferin; (**c**) free FITC group; (**d**) BHT group; (**e**) control group; (**f**) *ex vivo* images of the heart, liver, spleen, lung, kidney and brain.

*In vivo* imaging analysis: We studied the *in vivo* biodistribution of nanoparticles in mice, and fluorescence images were obtained at different time points post-injection. We found that FITC-nanometre aggregates were more enriched in the lung and brain than in other organs over time, with the maximum uptake at 2 h. The control group revealed that the majority of free FITC, free mangiferin and FITC with N-BHT was found in the liver and kidneys, suggesting that FITC-nanometre aggregates selectively targeted the lungs and brain. The *ex vivo* fluorescence images of excised tissues further confirmed a much higher accumulation of FITC-nanometre aggregates in the lung and brain compared to free FITC (Fig. 7).

## Discussion

BHT is a classic prescription, and its antipyretic effect results from synergistic interactions among all of the components in the formula. BHT not only inhibited fever in rabbits and reduced elevated TNF-α, IL-1β, IL-6, and CD8+ levels but also promoted a significant increase in the CD4+ level and CD4+/CD8+ levels^14^. As the principle ingredient of BHT, mangiferin provides pharmacological activities highly relevant to the therapeutic functions of the decoction. Recently, more attention has been paid to the conspicuous pharmacological activities of mangiferin; however, this compound shows poor solubility, mucosal permeability and bioavailability (merely 0.111 mg·mL^−1^), which seriously restricts its development and utilization.

For instance, when *Anemarrhenae Rhizoma* and *Cortex Phellodendri* are combined, the amount and rate of mangiferin absorption significantly decrease, but timosaponin from *Anemarrhenae* can obviously promote the *in vivo* absorption of mangiferin^15,16^. In contrast, the use of Chinese herbal medicines containing *Anemarrhenae* (as in BHT) increases the absorption of mangiferin, as evidenced in an everted gut sac model trial. Therefore, it is important to increase the solubility and bioavailability of these compounds in pharmaceutical processes to meet the preparation requirements and demand for clinical applications. In our paper, it is demonstrated by means of high-speed centrifugation coupled with dialysis and TEM that NAs exist in BHT. The results of the antipyretic experiment showed that N-BHT was more effective than the liquid and sediment of the BHT administered to the treatment groups. In particular, N-BHT was easily ingested by cells and targeted the brain and lungs, which often leads to fatal inflammation in the form of a high fever. Thus, it is reasonable to predict that antipyretic effects are associated with these nanoparticles.

Nanoparticles have been detected in Ma Xing Shi Gan Tang (MXSGT), and the formation of MXSGT nanometre aggregates may rely on the action of amphiphilic molecules (ephedrine and pseudoephedrine), which could interact with other molecules via hydrophobic or ionic interactions to form nanometre aggregates^17^. However, mangiferin and neomangiferin are not amphipathic molecules as ephedrine and pseudoephedrine are, so why they are loaded in nanometre aggregates? Why the nanometre aggregates formed and what forms of mangiferin and neomangiferin existed are very interesting questions. We have a hypothesis. Their absorption may occur through cooperation of the chemical constituents in *Rhizoma Anemarrhenae*, *Radix Glycyrrhizae*, japonica rice and gypsum. Combined with the characterization results and the results of related experiments, we surmised the formation mechanism of BHT nanoparticles. It is generally acknowledged that saponins from liquorice are promising solubilizers in Chinese herbal decoctions^18^. Reports have also shown that glycyrrhizin and its derivatives have a significant solubilization effect on saikosaponin and saikosaponian^19^. The solubility of mangiferin and neomangiferin was enhanced when *Rhizoma Anemarrhenae* and *Radix Glycyrrhizae* were boiled together. At the same time, boiling japonica rice generated many small granules with a water film containing abundant polysaccharides, which can be hydrolysed to monosaccharides and oligosaccharides. The chemical constituents would be embedded or adsorbed in the granules. Therefore, only the smaller granules, which ranged from 50 to 500 nm nanoparticles, would not be coagulated by the water film and electric double layer. Moreover, inorganic ions in gypsum, Ca^2+^, Mg^2+^, and Zn^2+^, act as zeta potential modifiers and play a critical role in sol stability to form an electric double layer. Neomangiferin and mangiferin were loaded in these nanoparticles. This finding may be a new description of the antipyretic mechanism of BHT and even lead to the development of an approach to finding new TCMs.

Naturally occurring nanometre aggregates were successfully separated from a decoction of a classic Chinese herbal medicine (BHT) with dialysis and high-speed centrifugation. Neomangiferin and mangiferin have been demonstrated to be associated with the nanometre aggregates, which may have a profound influence on the assimilation, delivery and bioactivities of BHT. Our approaches and discoveries may inspire studies on the interaction of active phytochemicals in herbal decoctions and the corresponding pharmacological implications. However, if the nanometre aggregates in BHT will be used as a new preparation, many studies on the methods of separation, purification, security and stability need to be carried out^20^.

## Methods

### Preparation of the dialysis bag

A 20 cm dialysis bag (Biosharp Biotechnology Co., Ltd.) with a 3500 molecular weight cut-off and 36 mm diameter was boiled for 10 min in ethylenediaminetetraacetic acid (EDTA) with bicarbonate, accurately adjusting the pH to 8. The dialysis bag was rinsed with distilled water to remove impurities. The above operations were repeated 2 times.

### High-speed centrifugation coupled with dialysis

**Figure 8:**
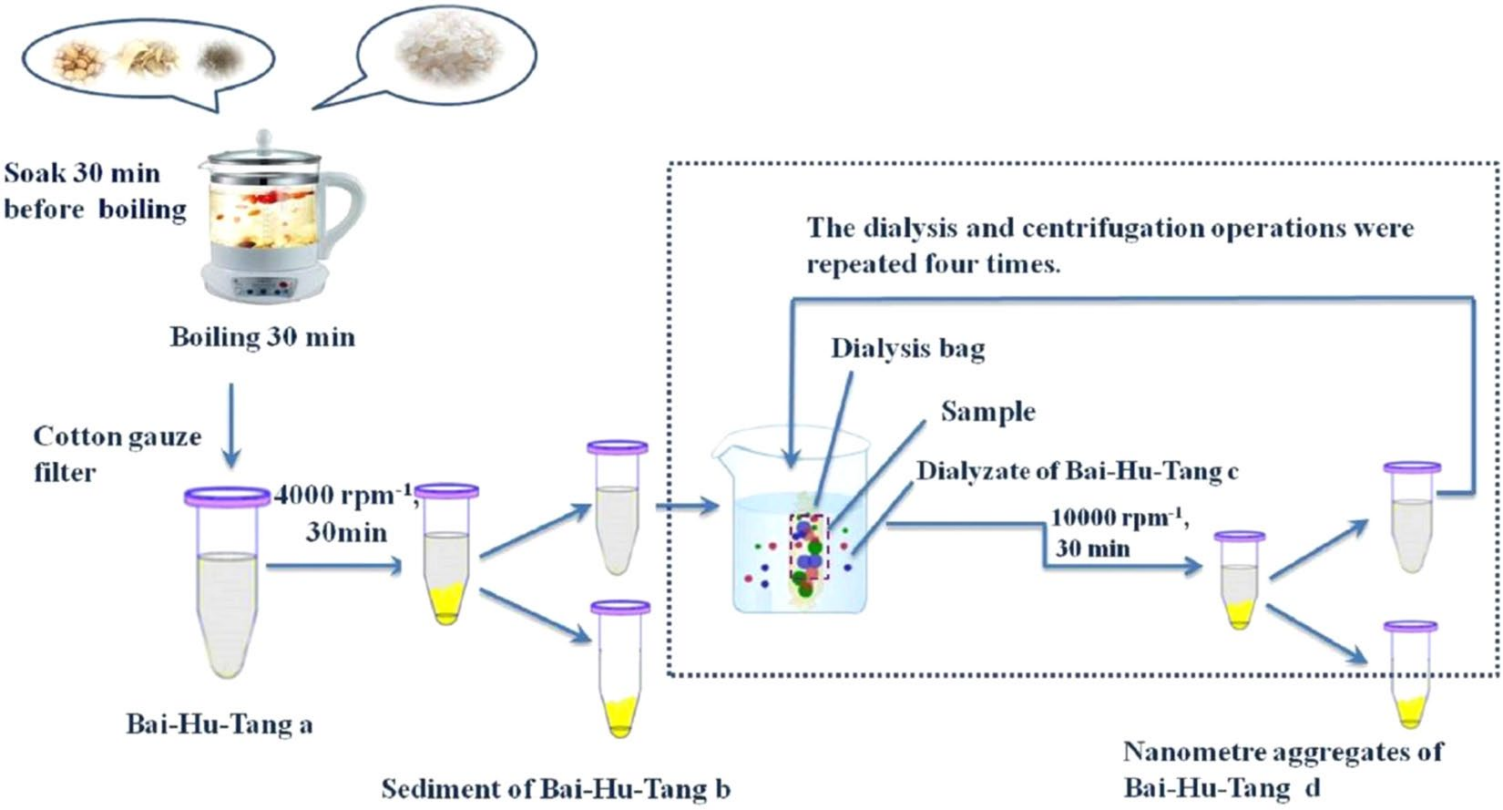
Isolation of nanometre aggregates from BHT by high-speed centrifugation and dialysis.

BHT (I) was centrifuged at 4000 rpm for 30 min; then, the supernatant was filtered through filter paper and a 0.45 μm membrane, sequentially. The filtrate was sealed in a 20 cm dialysis bag to constitute a unit. The unit was immersed in 200 mL of water in a beaker, which was placed on a constant-temperature vibrating water bath (Great Wall Material Industry and Trade Co., Ltd., Zhenzhou) and then dialyzed and separated at 120 rpm·min^−1^ for 30 min. Eventually, the liquid in the unit was removed by centrifugation at 10000 rpm·min^−1^ for 30 min (Xiang Yi H-2050R centrifuge). The dialysis and centrifugation operations were repeated four times. Through the above operations, the sediment of the Bai-Hu-Tang decoction (S-BHT, II), dialysate of the Bai-Hu-Tang decoction (D-BHT, III), and nanometre aggregates of the Bai-Hu-Tang decoction (N-BHT, IV) were acquired (Fig. 8).

### Transmission Electron Microscopy (TEM) and Particle Size Analyser

A drop of part IV was placed on a copper grid coated with carbon film and air-dried. Transmission electron microscopy (TEM, JEM2100, Japan) was performed to observe the morphology and analyse constituent elements. The particle size distribution was determined by a Malvern laser particle size analyser 2000. The stability of Part IV was also observed at 8, 16, and 24 h in the study.

### Reversed-phase high-performance liquid chromatography (RP-HPLC)

The analysis of multiple components occurred on a C18 column in an HPLC system (Thermo, Ultimate 3000). The mobile phase consisted of 25 mM dibasic potassium phosphate (solvent A) and acetonitrile (solvent B) with the following gradient elution program: 10 → 28 (A:B, v/v) at 0–18 min, 28 → 33 (A:B, v/v) at 18–20 min, and 33 → 39 (A:B, v/v) at 20–26 min. The appropriate concentration of the sample solution was selected, and the injection volume was 10 μL. The absorbance of the eluate was monitored at 257 nm. The flow rate was 1 mL·min^−1^, and the temperature of the column oven was set at 30 °C.

Primary stock solutions of neomangiferin (0.2 mg·mL^−1^), mangiferin (0.3 mg·mL^−1^), glycyrrhizic acid (0.1 mg·mL^−1^) and ammonium glycyrrhizinate (0.1 mg·mL^−1^) were prepared in methanol according to their solubility. The calibration curves were prepared with seven concentration levels, and the dilution factors were injected in different volumes of 0.2 μL, 0.5 μL, 1 μL, 2 μL, 4 μL, 8 μL, 10 μL, 12 μL, 14 μL, 16 μL and 20 μL.

Peaks were identified by comparing their retention time (tR) and UV spectra with those of standard compounds (neomangiferin, mangiferin, glycyrrhizic acid and ammonium glycyrrhizinate). Phases I~IV were condensed to 25 mL. Corresponding amounts (2 mL) of I~IV were suspended in methanol (8 mL) and placed in an ultrasonic bath (SB-5200D, Ningbo Scientz Biotechnology Co. Ltd.) for 10 min. Subsequently, the different phases were centrifuged at 2000 rpm·min^−1^ for 10 min, and the supernatants were subjected to RP-HPLC analysis.

### Temperature measurement and fever induction

Rabbits were purchased from the laboratory animal centre of Heilongjiang University of Traditional Chinese Medicine, Certificate No. SCXK(Hei)2008-004 (Heilongjiang, China). The temperature, humidity, and light conditions in the rabbits’ environment were kept constant, with food and water provided ad libitum. All rabbits were acclimated in the laboratory for at least one week prior to the experiment. Before testing, the animals were fasted overnight with free access to drinking water. All animal experiments were carried out in accordance with the Guidelines for the Care and Use of Laboratory Animals and were approved by the Animal Ethics Committee of Heilongjiang University of Chinese Medicine (No. 2016101601).

The rabbits were fixed in a prone position, and the basal Tb was monitored via an MC-347 soft electronic clinical thermometer inserted approximately 4 cm into the rectum. This procedure was performed at least twice in three days prior to the experiments to avoid rectal temperature changes after handling. The Tb of each rabbit was recorded as an average of the Tb over a period of 30 min prior to administration and fever induction. Animals with a Tb below 38 °C or above 40 °C were not used in any experiments. All the animals were used only once in this experiment.

Intravenous administration of LPS (20 μg·kg^−1^) elicited a biphasic thermal change in body temperature beginning 0.5 h after injection. The first thermal phase of the LPS response could characterize Yang-ming-re-zheng (in traditional Chinese medicine theory), while the second thermal phase always resulted in weakness and spirit depression^21^. Forty-two rabbits were randomly divided into seven groups, each with an identical number of rabbits (n = 6). One of the groups served as the ‘normal’ group, whereas the other 6 groups were subjected to the fever induction model. A febrile response was induced and monitored over time. Briefly, the Tb was immediately measured after rabbits received an injection of LPS in sodium chloride solution (20 μg·mL^−1^) in a marginal ear vein. Then, every 0.5 h, the temperature was measured once, and these measurements continued for 5 h. One measurement once was taken at every time point, and the values displayed were recorded.

The dose of the different dispersion phases of BHT was according to their content in the raw recipe. The grouping was as follows: normal group (normal), model group (NS, normal saline group), promethazine hydrochloride group (PH, positive control group), BHT group (BHT), sediment of BHT group (S-BHT), nanometre aggregates of BHT group (N-BHT), and dialysate of BHT group (D-BHT). The PH group was treated by gavage injection, and the treatment solution or normal saline was instilled into the colons of rabbits in the other experimental groups or control group, respectively, 0.5 h before LPS injection. The level of febrile response was expressed as the change in temperature from the basal temperature (°C), where the difference is expressed as ΔT (ΔT = measurement temperature-Tb).

### Interleukin levels

Five hours after the LPS injection, 2.0 mL blood samples were obtained from the ear artery with a disposable blood collector. The rabbits were killed immediately. The blood samples were transferred into aseptic tubes and centrifuged at 3000 rpm·min^−1^ for 10 min to obtain serum. The serum samples were kept at −20 °C for use in biochemical assays to determine IL-6, IL-1β, and TNF-α levels by commercially available ELISA kits according to the manufacturer’s protocol.

#### Cellular uptake

Fluorescence microscopy. The NAs of BHT were dialyzed at pH 9.0 (Na_2_CO_3_ aq.) overnight. FITC (1.0 mg) dissolved in water (0.5 mL) was added to the NAs of BHT, followed by magnetic stirring in darkness at 4 °C for 8 h to form nanometre aggregates associated with FITC. Caco-2 cells (purchased from Shanghai fu heng Biotechnology Co., Ltd.) were seeded at a density of 5.0 × 10^5^ cells on 24-well plates and maintained until the cells were adhered. Then, 0.1 mL of FITC-NAs were added to 0.5 mL of HBSS. FITC alone was the control group. Caco-2 cells were treated with the above samples and incubated at 37 °C and 5% CO_2_ for 24 h. The cell monolayers were rinsed three times with pre-warmed PBS (pH 7.4) and observed using an Olympus DP-71 digital camera (Olympus, Center Valley, PA).

Flow cytometry. To evaluate the cellular uptake rate, recording images is not sufficient. In this study, we also determined the uptake rate by flow cytometry (BD Accuri C6 flow cytometer, US). Briefly, Caco-2 cells were seeded at a density of 5.0 × 10^5^ cells on 24-well plates and were treated with the above samples for 2 h. After washing with PBS, the cells were digested with a solution of 0.25% trypsin and 0.02% EDTA for 3 min after washing with PBS (pH = 7.4). The samples were centrifuged for 5 min at 1000 rpm. The cells were redissolved in 2 mL of PBS and analysed by flow cytometry.

### *In vivo* imaging analysis

Mice were purchased from the laboratory animal centre of Heilongjiang University of Traditional Chinese Medicine, Certificate No. SCXK(Hei)2013-0004 (Heilongjiang, China). To evaluate the affinity towards different organs and the targeting ability of the nanometre aggregates, FITC-NAs were intravenously administered into the first group at a dose of 0.1 mg·kg^−1^. Normal saline (0.9%) was administered as a blank control. The other groups were given free FITC, free mangiferin and FITC with BHT as negative controls. FITC fluorescence was measured at excitation/emission wavelengths of 490/520 nm. Finally, the mice were sacrificed, and the heart, liver, spleen, lung, kidneys and brain of each mouse were removed to measure the individual fluorescence intensities.

### Statistical analyses

Each experiment was performed separately at least three times. The data were expressed as the mean ± standard deviation, and a statistical comparison between different groups was performed by a t-test. p < 0.05 indicates significance. Statistical analysis was conducted using SPSS 19.0.
